# Vectorial secretion of CTGF as a cell-type specific response to LPA and TGF-β in human tubular epithelial cells

**DOI:** 10.1186/1478-811X-10-25

**Published:** 2012-09-02

**Authors:** Jonathan Zuehlke, Astrid Ebenau, Bettina Krueger, Margarete Goppelt-Struebe

**Affiliations:** 1 Department of Nephrology and Hypertension, Friedrich-Alexander Universität Erlangen-Nürnberg, Loschgestrasse 8, Erlangen 91054, Germany; 2 Department of Cellular and Molecular Physiology, Friedrich-Alexander Universität Erlangen-Nürnberg, Waldstrasse 6, Erlangen, 91054, Germany

**Keywords:** Connective tissue growth factor, Primary human tubular epithelial cells, Transforming growth factor β, Lysophosphatidic acid, Vectorial secretion, Cell polarization

## Abstract

**Background:**

Increased expression of the pro-fibrotic protein connective tissue growth factor (CTGF) has been detected in injured kidneys and elevated urinary levels of CTGF are discussed as prognostic marker of chronic kidney disease. There is evidence that epithelial cells lining the renal tubular system contribute to uptake and secretion of CTGF. However, the role of different types of tubular epithelial cells in these processes so far has not been addressed in primary cultures of human cells.

**Results:**

Tubular epithelial cells of proximal and distal origin were isolated from human kidneys and cultured as polarized cells in insert wells. The pro-fibrotic stimuli lysophosphatidic acid (LPA) and transforming growth factor β (TGF-β) were used to induce CTGF secretion.

LPA activated CTGF secretion in proximal tubular cells when applied from either the apical or the basolateral side as shown by immunocytochemistry. CTGF was secreted exclusively to the apical side. Signaling pathways activated by LPA included MAP kinase and Rho kinase signaling. TGF-β applied from either side also stimulated CTGF secretion primarily to the apical side with little basolateral release.

Interestingly, TGF-β activation induced different signaling pathways depending on the side of TGF-β application. Smad signaling was almost exclusively activated from the basolateral side most prominently in cells of distal origin. Only part of these cells also synthesized CTGF indicating that Smad activation alone was not sufficient for CTGF induction. MAP kinases were involved in apical TGF-β-mediated activation of CTGF synthesis in proximal cells and a subset of epithelial cells of distal origin. This subpopulation of distal tubular cells was also able to internalize recombinant apical CTGF, in addition to proximal cells which were the main cells to take up exogenous CTGF.

**Conclusions:**

Analysis of polarized human primary renal epithelial cells in a transwell system shows that vectorial secretion of the pro-fibrotic protein CTGF depends on the cell type, the stimulus and the signaling pathway activated. In all conditions, CTGF was secreted mainly to the apical side upon TGF-β and LPA treatment and therefore, likely contributes to increased urinary CTGF levels in vivo. Moreover, CTGF secreted basolaterally may be active as paracrine pro-fibrotic mediator.

## Background

Connective tissue growth factor (CTGF, CCN2) is a secreted matricellular protein which has been associated with fibrotic diseases, often mediating the pro-fibrotic effects of transforming growth factor β (TGF-β) [1,2]. Elevated levels of CTGF have been described in conditions of tissue injury and inflammation, in different animal models of kidney injury and also in human biopsy specimens [3-5]. In the rat model of unilateral ureter obstruction (UUO) CTGF protein was increased prominently in tubular epithelial cells [6]. An increased number of cells expressing CTGF mRNA was observed in human biopsies specimens at sites of chronic tubulointerstitial damage, the majority of which co-expressed alpha-smooth muscle actin [7]. Thus, inflammatory and fibrotic situations seem to induce CTGF synthesis in interstitial as well as in tubular cells.

Urinary CTGF is a marker of chronic kidney disease such as progressive hypertensive nephrosclerosis [8], diabetic nephropathy [9,10] or chronic renal allograft injury [11,12]. It was assumed that urinary CTGF reflected increased synthesis of CTGF by glomerular and tubulointerstitial cells. Analysis of human kidney sections showed CTGF mRNA and protein in severely damaged human tubules but not in normal epithelial cells [7,13]. However, in a recent study, proximal tubular dysfunction was identified as major determinant of urinary CTGF excretion [14]. CTGF was detected in apical endocytic vesicles of proximal tubular cells in mice treated with recombinant CTGF, suggesting that under normal conditions CTGF is reabsorbed almost completely and thus not detectable in the urine unless reabsorption is impaired. Depending on the pathophysiological setting, tubular epithelial cells may thus be a source or sink for urinary CTGF.

In vivo, epithelial cells are polarized with structurally and functionally distinct basolateral and apical domains. Polarization of epithelial cells in vitro can be achieved by culturing the cells on transwell membranes where they have access to nutrients and growth factors from two sides. MDCK cells derived from distal tubular cells readily establish dense polarized monolayers characterized by high transepithelial electrical resistence (TEER), whereas cell lines obtained from proximal parts of the nephron show lower TEER in line with the higher capacity for paracellular transport of proximal nephron segments [15,16]. Correspondingly, epithelial cells lining the different parts of the nephron vary in their cell-cell adhesion proteins, tight junction proteins and cadherins [17]. Proximal tubular cells are the only epithelial cells in the human adult organism which express N-cadherin instead of E-cadherin as major cell-cell adhesion protein, which allows differentiation of cells of proximal and distal origin by immunocytochemistry [18,19]. CTGF secretion from polarized human tubular cells has not been addressed at all and the subtype of tubular cells responsible for CTGF secretion has not been identified. Furthermore, the direction of CTGF secretion to the apical or basolateral side is unknown, because thus far, CTGF synthesis has been analyzed only in non-polarized primary human tubular epithelial cells and epithelial cell lines [18,20-22].

While TGF-β is the most important pro-fibrotic stimulus, other mediators add to inflammatory and fibrotic reactions in the kidney as, for example, lysophosphatidic acid (LPA). LPA is released from platelets upon stimulation but is also produced from phosphatidylcholine by extracellular phospholipases upon cell damage [23]. By binding to several G-protein-coupled receptors it acts as pleiotropic mediator of multiple cellular effects [24]. In earlier studies, LPA was characterized as a growth and survival factor of proximal tubular cells [25] and related to inhibition of apoptosis and complement activation in reperfusion injury [26]. More recently activation of LPA1 receptors was reported in the UUO model of renal fibrosis, which was ameliorated upon LPA1 inhibition [27]. Along this line we have shown earlier that LPA is a potent inducer of CTGF in several cell types, among them mesangial cells and renal fibroblasts [28,29]. LPA as an activator of CTGF synthesis has not yet been investigated in primary tubular epithelial cells.

In this study, we addressed the question whether epithelial cells of proximal or distal tubular origin respond to the pro-fibrotic stimuli LPA and TGF-β by vectorial stimulation of CTGF synthesis and secretion. For this purpose, we used our recently established cell culture model of primary human tubular cells [18]. We show marked differences between both stimuli in terms of activated cells and route of application.

## Results

### Apical secretion of CTGF upon stimulation with LPA

**Figure 1:**
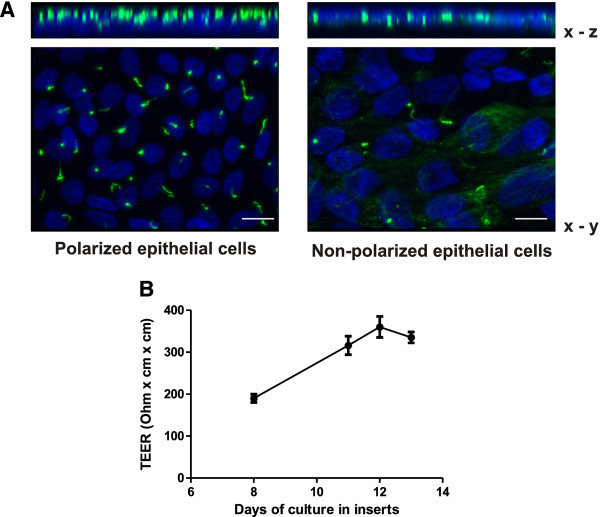
**Polarized culture of primary human tubular epithelial cells (hPTEC).A**: Freshly isolated hPTEC were cultured for 8 days in transwell inserts. Cilia were stained with acetylated tubulin and nuclei were visualized by Hoechst. For comparison, non polarized cells are shown in the right panel. Scale bar: 10 μm. **B**: hPTECs were cultured for 8 to 13 days in transwell inserts. Transepithelial electrical resistance (TEER) was measured in three cultures of one preparation. Data are means +/− SD.

Primary tubular cells were isolated from healthy parts of human kidney nephrectomies. Cells obtained from proximal or distal parts of the nephron were distinguished by the expression of specific cell-cell adhesion molecules, N-cadherin in proximal and E-cadherin in distal tubular cells as described earlier [18]. When the cells were cultured for at least 8 days on porous membranes in transwell inserts, they developed a polarized structure, visualized by the apical orientation of cilia above the nuclei identified by staining with an antibody against acetylated tubulin (Figure1A). For comparison, non polarized cells display cilia which do not extend above the cells. In these cells, part of the tubular network also consisted of acetylated tubulin (Figure1A). To show the integrity of the polarized monolayer, transepithelial electrical resistance (TEER) was determined using a Voltohmeter (EVOM). Values of 100 to 200 Ω x cm^2^ were obtained after 8 days and increased when the cells became denser (Figure1B). As expected, there was variability depending on the composition of the primary cells, resistance being higher in distal cells compared to proximal cells. Figure1B shows an example of a preparation consisting primarily of distal tubular cells. 

**Figure 2:**
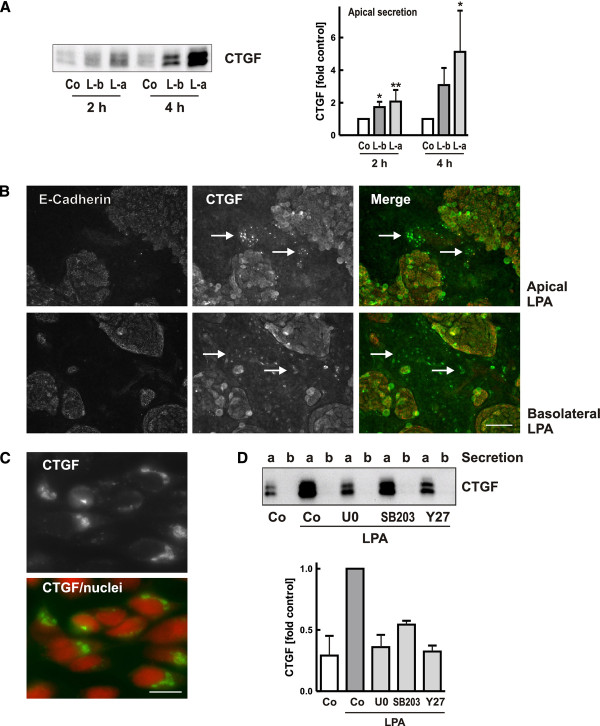
**Incubation with apical or basolateral LPA stimulated apical secretion of CTGF.A**: Polarized hPTEC were stimulated with LPA (10 μM) from the basolateral side (L-b) or from the apical side (L-a) for 2 and 4 h. CTGF was detected in the apical compartment. The graph summarizes data (means +/− SD) of 5 (2 h) and 3 (4 h) independent experiments. Values of secretion of control cells were set to 1 at each time point. * p < 0.05; ** p < 0.01, Dunnet’s multiple comparison test. **B**/**C**: Polarized hPTEC were stimulated with LPA (10 μM) from the apical or basolateral side for 1 h. CTGF and E-cadherin were visualized by immunocytochemistry. Nuclei were stained with Hoechst. Cells negative for E-cadherin represent proximal tubular cells. Newly synthesized CTGF was detectable in a perinuclear localization (C; nuclei colored in red; CTGF in green, scale bar: 10 μm). Arrows indicate examples of CTGF positive proximal cells. Scale bar: 50 μm. **D:** Polarized hPTEC were pre-incubated with the inhibitors (U0: U0106, 1 μM; SB203: SB203580 1 μM; Y27: Y27632, 10 μM) for 30 min and then stimulated with LPA (10 μM) from the apical and basolateral side for 4 h. Secreted CTGF was detected in the apical (a) and basolateral (b) compartment. The graph summarizes data obtained with two isolations. Apical secretion of LPA-stimulated cells was set to 1 in each experiment.

Polarized epithelial cells were stimulated with LPA (10 μM) from the apical or the basolateral side. Apical secretion of CTGF was rapidly induced within 2 to 4 h as shown by Western blotting (Figure2A), whereas basolateral CTGF secretion was below the detection limit (Figure2C). There was no significant difference between apical and basolateral application of LPA.

By immunocytochemical analysis intracellular CTGF is only visible when it is newly synthesized and transported in vesicles from the endoplasmic reticulum through the golgi [30]. Ongoing CTGF synthesis was detected by both, basolateral and apical application of LPA. CTGF expression was almost exclusively in cells of proximal origin, which lacked E-cadherin expression (Figure2B). Higher magnification showed the perinuclear localization of CTGF (Figure2C).

Using low molecular weight inhibitors we analyzed which signaling pathways contributed to LPA-mediated up-regulation of CTGF expression. Inhibition of p42/44 MAP kinase/ERK1/2 activation by U0106 and to a lesser degree inhibition of the activity of p38 kinase by SB203580, reduced LPA-induced secretion of CTGF. Furthermore, Rho kinases were involved as LPA-mediated induction of CTGF was prevented by Y27632, an inhibitor of Rho-kinase signaling (Figure2D).

### Basolateral and apical stimulation by TGF-β induced apical secretion of CTGF

**Figure 3:**
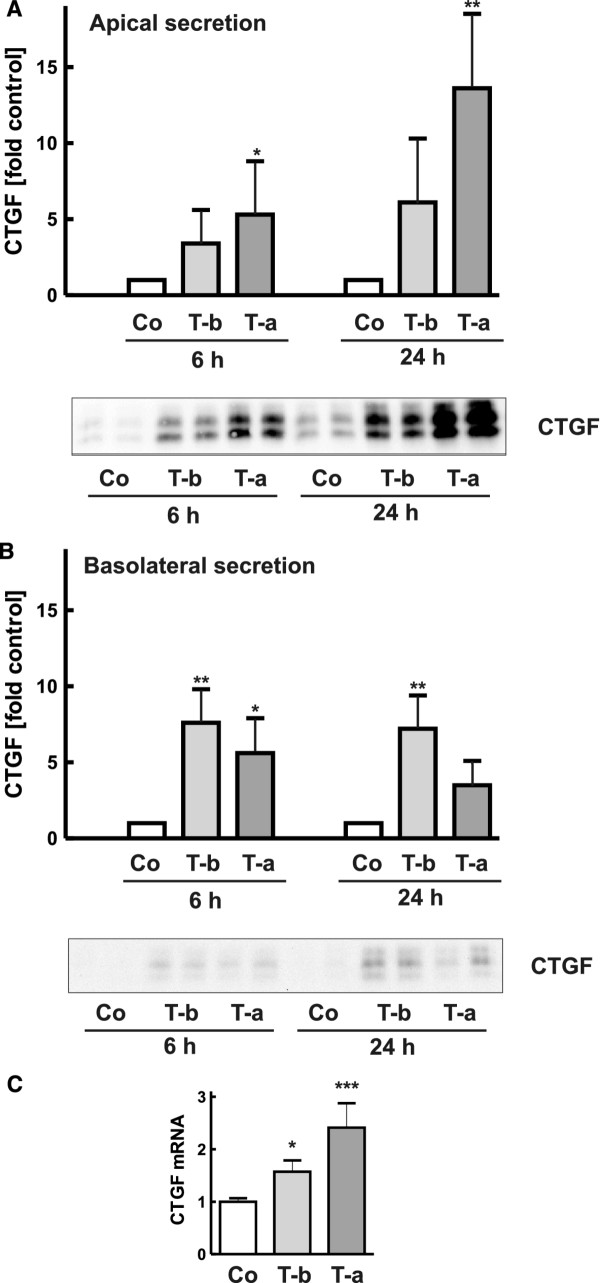
**Secretion of CTGF from polarized hPTEC stimulated with TGF-β.** Polarized hPTEC were stimulated with TGF-β for 6 and 24 h from the basolateral side (T-b) or from the apical side (T-a). CTGF was detected in the apical (**A**) or basolateral (**B**) compartment. The blots shown are from a representative experiment performed with duplicate inserts. The graphs summarize means +/− SD of 6 (A) and 3 (B) experiments with different hPTEC isolations. Expression of control cells in each experiment was set to 1. * p < 0.05, ** p < 0.01, Dunnet’s multiple comparison test. **C**: RNA was isolated from polarized cells treated with TGF-β for 3 h. CTGF mRNA expression was determined by real time quantitative RT-PCR and corrected for 18 S RNA. Data are means +/− SD of duplicate determinations of duplicate inserts. RNA in control cells was set to 1. * p < 0.05, ** p < 0.01, Dunnet’s multiple comparison test.

Secretion of CTGF induced by TGF-β was detectable after 6 and 24 h and was thus slower than upon stimulation with LPA (Figure3). There was a marked difference in the amount of CTGF retrieved from the apical and basolateral compartments, apical secretion being much higher than basolateral secretion. TGF-β stimulated apical CTGF secretion when applied to the apical or basolateral side of the cells, although apical application was more effective (Figure3A). Basolateral secretion was very low, irrespective of TGF-β application from the apical or basolateral side and was not detectable in all experiments (Figure3B). As a matricellular protein CTGF binds to extracellular matrix proteins but may also attach to plastic. To prevent loss of CTGF from the supernatants, experiments were also performed in the presence of heparin, a binding protein for CTGF. The total amount of CTGF detectable in the supernatants was increased whereas the proportions of CTGF secretion were not altered (data not shown). To further characterize regulation of CTGF expression mRNA was isolated from cells cultured in inserts and analyzed by realtime quantitative PCR (Figure3C). Stimulation with apical or basolateral TGF-β increased CTGF mRNA levels in line with transcriptional regulation of CTGF expression.

**Figure 4:**
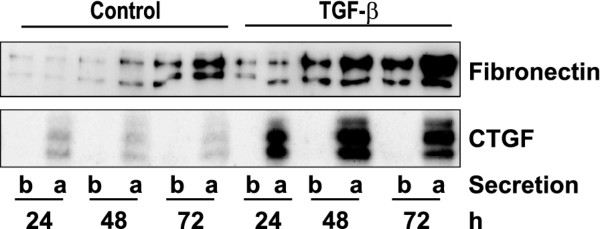
**Secretion of fibronectin.** Polarized hPTEC were stimulated with TGF-β from the basolateral and from the apical side for 24 to 72 h. Secreted CTGF and fibronectin were detected in the basolateral (b) or apical (a) compartment. The blot is representative of 3 experiments with different hPTEC isolations. In the experiment shown CTGF was below the detection limit in the basolateral compartment.

As TGF-β is a potent inducer of extracellular matrix proteins, we additionally analyzed fibronectin secretion. In contrast to CTGF secretion, released fibronectin was not detectable until 24 h after induction by TGF-β. Moreover, there was a considerable release of fibronectin to the basolateral side in all experiments (example shown in Figure4). Quantification of fibronectin in the cell culture supernatant was variable most likely due to the fact that secreted fibronectin is not entirely soluble but forms a fibrillar network attached to the cells.

### Predominant activation of distal tubular cells by TGF-β

**Figure 5:**
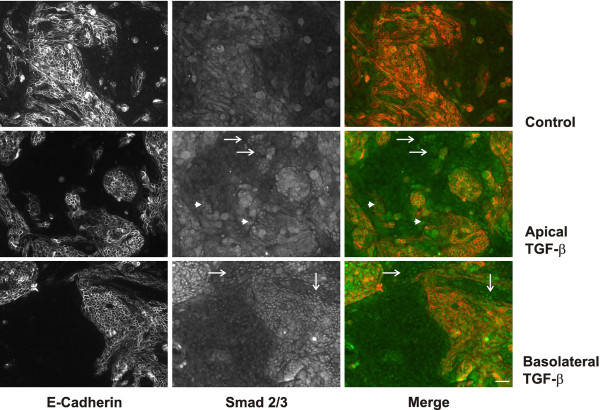
**Preferential activation of Smad2/3 in polarized hPTEC of distal origin.** Polarized hPTEC were treated with TGF-β for 1 h from the apical or basolateral side. E-cadherin (red) and Smad2/3 (green) were detected by immunocytochemistry. Cells negative for E-cadherin represent proximal cells. Upon activation with TGF-β, Smad2/3 was concentrated in the nuclei of proximal and distal cells, respectively. Arrows indicate nuclear Smad2/3 in proximal E-cadherin negative cells and arrow heads indicate nuclear Smad2/3 in distal weakly E-cadherin positive cells. Scale bar: 50 μm.

Immunocytochemical analyses were performed to distinguish effects of TGF-β on cells of proximal and distal origin. Activation of Smads is one of the major signaling pathways of TGF-β and was detected as nuclear translocation of Smad2/3 after 1 h incubation. In Figure5, Smad2/3 translocation was analyzed and correlated to E-cadherin double staining identifying cells of distal origin. In control cells, Smad 2/3 was located in the cytosol. Treatment with TGF-β from the apical side activated translocation of Smad 2/3 only in few cells. Part of them were E-cadherin negative and thus of proximal origin (arrows in Figure5). Other responding cells showed weak E-cadherin staining (arrowheads, Figure5) which marked them as distal tubular cells. By contrast, TGF-β stimulation from the basolateral side quantitatively activated Smad2/3 translocation in all E-cadherin positive cells and also some proximal cells (examples indicated by arrows in Figure5, lower panel). Location of Smad2/3 in the nucleus of proximal cells was confirmed by co-staining of N-cadherin (data not shown).

**Figure 6:**
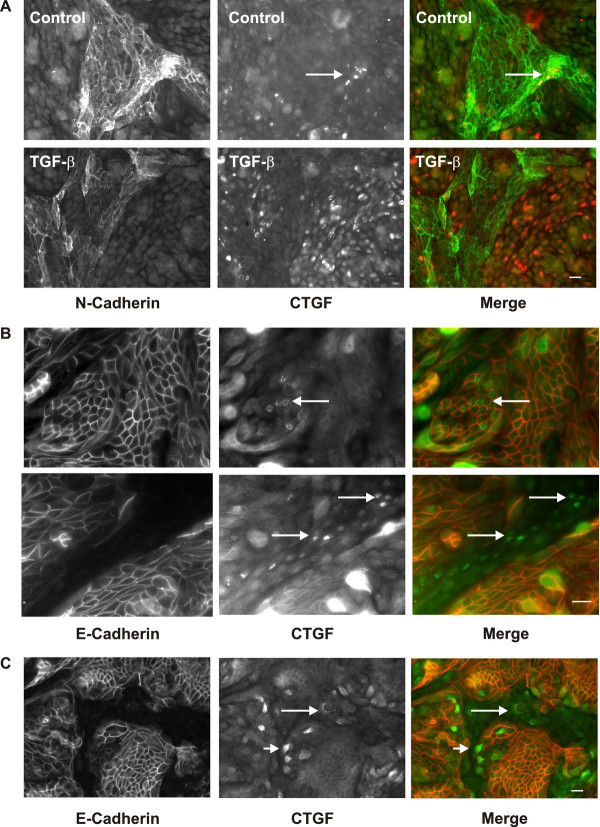
**Accumulation of intracellular CTGF upon stimulation with basolateral TGF-β.A**: hPTEC were polarized and then stimulated with TGF-β for 1 h from the basolateral side. N-cadherin (green) and CTGF (red) were detected by immunocytochemistry. Control cells showed CTGF expression only in clustered proximal tubular cells (arrow, upper panels). Treatment with TGF-β from the basolateral side activated CTGF primarily in distal tubular cells (lower panels). Scale bar: 20 μm. **B**: Higher magnification of an experiment performed as in A with cells stimulated with TGF-β from the basolateral side. E-cadherin (red) and CTGF (green) were detected by immunocytochemistry. Arrows indicate examples of positive cells. Scale bar: 20 μm. **C**: Polarized hPTEC were treated with brefeldin A (10 μM) for 30 min and then were incubated with TGF-β from the apical side for 2 h. E-cadherin (red) and CTGF (green) were detected by immunocytochemistry. Arrows indicate examples of CTGF positive proximal cells, which were negative for E-cadherin; arrow heads indicate examples of CTGF positive distal cells, which weakly express E-cadherin. Scale bar: 20 μm.

CTGF is a secreted protein and therefore difficult to detect by immunocytochemistry within cells. In control cells, CTGF synthesis was often detected in proximal epithelial cells which formed irregular densely packed structures (Arrows in Figure6A, upper panel). Upon basolateral stimulation with TGF-β CTGF was primarily, but not exclusively, detected in distal, E-cadherin-positive, tubular epithelial cells (Figure6A, lower panel). In contrast to the general activation of Smad2/3 in distal tubular cells, induction of CTGF expression was restricted to individual distal cells as shown by higher magnification (Arrows in Figure6B, upper panel). Although only occasionally, we observed CTGF secretion also in proximal epithelial cells (Arrows in Figure6B, lower panel).

Stimulation with TGF-β from the apical side for 1 and 2 h did not lead to accumulation of CTGF in intracellular vesicular structures, suggesting a rapid release mechanism. To interfere with CTGF secretion polarized cells were treated with brefeldin A which disrupts Golgi structures and thus inhibits transport of secreted proteins. In the presence of brefeldin A TGF-β applied to the apical side of the cells induced CTGF accumulation primarily in a subset of distal tubular cells expressing low levels of E-cadherin (Figure6C, arrow heads), and also in individual proximal cells (Figure6C, arrows).

Taken together, basolateral application of TGF-β induced CTGF synthesis primarily in distal tubular cells whereas apical application activated proximal cells and a subset of weakly E-cadherin positive distal cells.

### MAP kinases are essential for apical TGF-β -mediated secretion of CTGF

**Figure 7:**
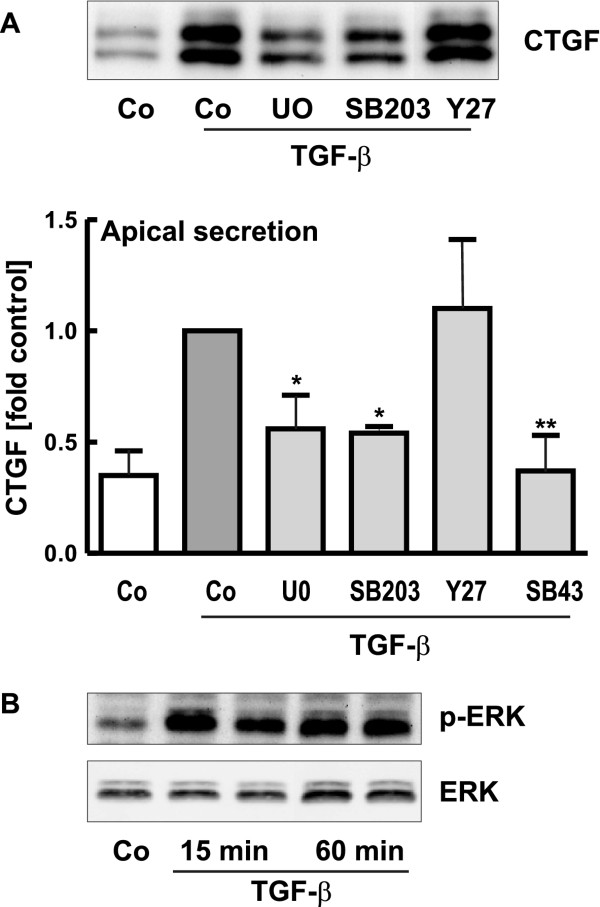
**Role for MAP kinases in apical TGF-β-mediated secretion of CTGF.A**: Polarized hPTEC were incubated with inhibitors (U0: U0106, 1 μM; SB203: SB203580, 1 μM; Y27: Y27632, 10 μM; SB43: SB431542: 10 μM) for 30 min and then stimulated with TGF-β from the apical side for 6 h. CTGF secretion was detected in the apical compartment. The graph summarizes means +/− SD of 3 independent experiments. Secretion of CTGF in TGF-β-stimulated cells was set to 1 in each experiment. ** p < 0.01, * p < 0.05 compared to TGF-β-stimulated cells, Dunnet’s multiple comparison test. **B**: Polarized hPTECs were stimulated in duplicate with TGF-β for 15 and 60 min from the apical side. Protein was isolated from polarized cells. ERK1/2 and phospho-ERK1/2 were detected by Western blotting.

The poor activation of Smad translocation by apically applied TGF-β suggested additional non-canonical signaling pathways being involved in CTGF induction. We pre-incubated hPTEC with the indicated inhibitors and then stimulated the cells with TGF-β from the apical side. Inhibition of Rho kinases did not significantly reduce CTGF induction, whereas inhibition of ERK1/2 by UO106 and p38 kinase by SB203580, almost completely prevented apical TGF-β-mediated induction of CTGF secretion (Figure7A). Furthermore, CTGF induction was completely prevented, when the TGF-β receptor I (Alk-5) was inhibited by SB43152 indicating that the cells express TGF-β receptors also in the apical membrane. To support a role for ERK1/2 in TGF-β signaling, activation of ERK1/2 was determined in cellular homogenates of cells cultured in inserts. As shown by Western blot analysis, ERK1/2 was phosphorylated when TGF-β was applied apically (Figure7B).

### Uptake of apical CTGF by proximal tubular epithelial cells

**Figure 8:**
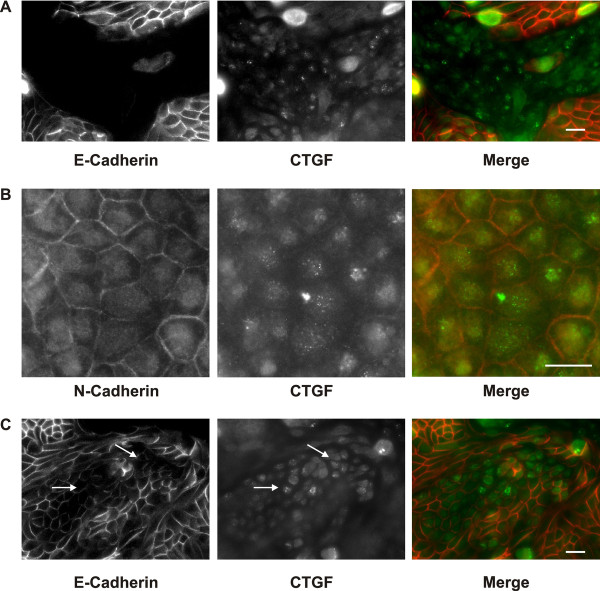
**Uptake of extracellular CTGF from the apical side.A**: Polarized hPTEC were treated with recombinant CTGF (10 ng/ml) for 15 min from the apical side. Cells were stained for E-cadherin (red) and CTGF (green). CTGF immunoreactivity was detected primarily in E-cadherin negative, proximal cells. Scale bar: 20 μm. **B**: Cells were treated as in A. N-cadherin positive cells (red) are shown at higher magnification. CTGF (green) is detectable in vesicular structures. Scale bar: 20 μm. **C**: Cells were treated as in A. Cells weakly positive for E-cadherin (red) showed CTGF (green) immunoreactivity, whereas distal cells with strong E-cadherin cell-cell contacts were negative. Arrows indicate cells positive for CTGF. Scale bar: 20 μm.

CTGF has been reported to be taken up by mouse proximal tubules in vivo [14]. Therefore, we analyzed uptake of exogenous CTGF in polarized hPTEC. Polarized cells were incubated with recombinant human CTGF applied to the apical or basolateral side. Within 15 minutes, apical CTGF was detectable in intracellular vesicles. Uptake was confined to N-cadherin expressing proximal tubular cells (Overview in Figure8A, details at higher magnification Figure8B). Furthermore, uptake was detectable in a subset of weakly stained E-cadherin positive cells (Figure8C), which did not express N-cadherin (data not shown). Cells with strong E-cadherin positive cell-cell contacts did not take up exogenous CTGF. No vesicular CTGF uptake was detectable when recombinant CTGF was applied to the basolateral compartment. 

## Discussion

In this study we show that CTGF is secreted from different types of polarized epithelial cells derived from the human renal tubular system. We provide evidence that CTGF secretion is specifically regulated depending on the cell type, the stimulus and the side of application. Proximal tubular cells were the main target cells of LPA stimulation and Rho kinase signaling was activated in these cells. By contrast, TGF-β primarily stimulated different types of distal tubular epithelial cells. Basolateral TGF-β activated Smad signaling whereas MAP kinases were essential for apical activation of CTGF secretion by TGF-β.

Epithelial cells derived from human kidneys are functionally and structurally heterogeneous depending on their origin along the renal tubular system. This is also reflected in terms of epithelial barrier function and, at the cellular level, tight junctions. Renal proximal tubules are characterized as ‘leaky’ nephron segments with paracellular transport of NaCl, which may in part relate to the expression of claudin-2 [31,32]. Claudin-2 has also been shown to be involved in transepithelial resistance determined in different types of MDCK cells [33]. Primary cells obtained from healthy sections of human tumor nephrectomies differ in their composition as cells are obtained from various parts of the nephron. This was reflected in our measurements of transcellular resistance of the polarized cells which varied depending on the cellular composition. Preparations which consisted almost exclusively of cells of distal origin showed higher resistance than preparations which contained a higher proportion of proximal cells.

By immunocytochemistry, human cells of proximal and distal origin can be distinguished by their expression of different cell-cell adhesion proteins [18]. Morphologically, E-cadherin positive cells could be further subdivided in cobble-stone like cells which showed a strong E-cadherin immunoreactivity, and more elongated cells with less intense staining for E-cadherin. Studies are ongoing to further characterize the origin of these cells, because they markedly differed in their response to TGF-β as outlined in detail below. Proximal tubular cells uniquely express the mesenchymal cell-cell adhesion molecule N-cadherin. These cells appeared to be less polarized than distal cells, and their adherence to membranes is less tight. In the coculture system of primary cells proximal cells were surrounded by distal cells and thus exposed to mechanical stress. Proximal cells tended to form multilayered structures, which often stained positive for CTGF in line with the known sensitivity of CTGF expression to mechanical stimulation [34,35].

Proximal tubular cells thus seem to be more susceptible to stimuli which modulate the actin cytoskeleton, be it by mechanical stress or by stimuli such as LPA, which activates Rho-Rho kinase signaling and modulates actin stress fibers [24]. We have shown earlier that induction of CTGF is sensitive to Rho-Rho kinase signaling in mesenchymal cells and that alterations in the actin cytoskeleton may induce CTGF expression by modulating G-actin and MAL/SRF dependent transcription in renal fibroblasts and endothelial cells, respectively [28,36,37]. By contrast, regulation of MAL/SRF-mediated gene regulation was mediated by Rac1 signaling rather than Rho-Rho kinase signaling in MDCK cells activated by calcium deprivation [38]. In polarized hPTEC, inhibition of Rho kinase signaling affected LPA-induced CTGF up-regulation but not TGF-β-mediated induction. This may be due to different signaling pathways activated by both stimuli and by the cell types activated. LPA activated almost exclusively N-cadherin positive proximal tubular cells.

Secretion of CTGF was clearly favored to the apical side of the cells. Thus far, rather little is known about the molecular mechanisms regulating CTGF secretion. Comparison of CTGF secretion with the secretion of fibronectin indicated that the apical secretion of CTGF was a regulated vectorial process which differed from the secretion of fibronectin, the basolateral secretion of which was much more pronounced. Other secreted proteins such as e.g. metalloproteinases were detected comparably in the apical and basolateral compartment of the transwell cultures (data not shown) further endorsing the specificity of CTGF secretion.

Based on the data obtained with LPA, it was clear that proximal tubular epithelial cells secreted CTGF to the apical side. However, as long as the molecular mechanisms of CTGF secretion have not been deciphered, we cannot rule out that other stimuli may activate also basolateral secretion of CTGF from proximal tubular cells.

LPA stimulated CTGF expression applied from both sides. In line with these data CTGF receptors have been characterized in both, apical and basolateral membranes of polarized gastrointestinal Caco-2 cells [39]. By contrast, TGF-β was reported to activate Caco-2 cells and MDCK cells only when applied to the basolateral side [40-42]. However, localization of TGF-β receptors seems to vary between different epithelia. Even with certain MDCK cells (subtype MDCKII) apical activation by TGF-β has been reported [43]. Apical localization of TGF-β receptor I was also reported in porcine vas deferens epithelium [44]. In the kidney, Wang et al. had provided evidence for apical expression of TGF-β receptors in a subset of rat cortical collecting duct cells, and showed in vitro reactivity of mouse proximal tubular cells and renal inner medullary cell lines (mIMCD-3 cells) upon apical stimulation with TGF-β [45]. In our experiments, TGF-β was clearly active when applied from the apical side in a TGF-β receptor I-dependent manner. Immunocytochemical analyses revealed that in addition to proximal tubular cells only a subset of distal cells reacted to apical TGF-β, characterized by low expression of E-cadherin. Further studies are necessary to define the origin of these cells which obviously differ from MDCK cells.

An additional level of complexity arises from the diversity of TGF-β receptor signaling, which implies Smad dependent and independent pathways (summarized in [46]). Furthermore, TGF-β signaling depends on interactions of its recptors with other membrane-bound proteins including growth factor receptors and integrins [47,48]. In our studies, apical TGF-β hardly activated the canonical Smad pathway which was detected upon basolateral application and may be essential for TGF-β secretion as detected in MDCK cells. In MDCKII cells, apical treatment with TGF-β increased transepithelial resistance, which was prevented by inhibition of p38 and ERK1/2 signaling [43]. In line with those studies, we showed phosphorylation of ERK1/2 upon apical application of TGF-β related to increased CTGF synthesis. A role for active ERK1/2 in CTGF synthesis in non-polarized epithelial cells was also described in epithelial cells of the eye [49] and in proximal tubular cell lines HKC-8 [21,22]. Activation N-Ras GTPase has been implicated in ERK-mediated CTGF induction in HKC-8 cells [22]. Furthermore, evidence was provided for direct phosphorylation of ShcA proteins by TGF-β receptor I [50]. Whether these or other signaling pathways are also involved in the apical induction of CTGF in primary human tubular cells remains to be investigated.

Basolateral stimulation of tubular epithelial cells by TGF-β rapidly activated Smad signaling in all distal tubular cells and also part of the proximal cells. By contrast, only distinct cell populations stained positive for intracellular CTGF at any given time point after TGF-β stimulation. This may in part be due to the fact that intracellular CTGF can only be detected when it accumulates in the secretory pathway. However, more likely it reflects the fact that activation of the Smad pathway is not sufficient for CTGF synthesis and secretion. For instance, induction of CTGF by TGF-β in epithelial cells cultured in dishes was strongly dependent on cell density, whereas Smad translocation was density-independent [20]. The signaling pathways which regulate density-dependent CTGF synthesis are not yet known but may also modulate CTGF expression in polarized cells.

Cellular uptake of CTGF has been observed in different cell types, mesangial cells [51], fibroblasts [52], chondrocytes [53] or endothelial cells (Muehlich, Goppelt-Struebe, unpublished), and was also shown in vivo in proximal tubular cells in mice upon CTGF injection [14]. Our experiments confirmed uptake of exogenous CTGF in proximal cells. In addition, we observed CTGF uptake in the subset of E-cadherin positive cells, which also secreted CTGF upon stimulation with apical TGF-β as discussed above.

It was interesting to note that upon stimulation with TGF-β, CTGF was not exclusively secreted to the apical side but also detectable at the basolateral side. Extrapolating to the in vivo situation, this may imply that the secreted CTGF is not lost in the urine but may be potentially active as a paracrine mediator. This aspect is supported by in vivo studies showing a role for tubular CTGF in the model system of remnant kidney disease in TGF-β1 transgenic mice [54]. Activated epithelial cells may thus contribute to the increased CTGF plasma levels detected in chronic kidney disease also in humans [1-3]. Furthermore, the strong apical secretion of CTGF suggests that also in vivo, activated tubular cells may have a share in urinary CTGF levels not only by uptake of filtered CTGF but also by secretion. Whether or not urinary CTGF has a functional role by acting on further distal tubular cells, remains to be established. Thus far, the biological effects of CTGF on distal tubular cells have not yet been investigated.

## Conclusions

In this study we provide evidence that non transformed polarized human epithelial cells secrete the pro-fibrotic protein CTGF in a cell-type and stimulus-specific manner. Furthermore, CTGF secretion was distinguishable from other secreted proteins indicative of specific molecular mechanisms regulating vectorial secretion. Most interestingly, CTGF was not only secreted but also taken up by proximal tubular cells. Approximating the in vivo situation, our model system is thus suitable for further investigation of secretion and uptake of proteins that are relevant markers for renal injury.

## Methods

### Materials

DMEM/Ham’s F12 medium was purchased from Biochrom AG (Berlin, Germany), DMEM medium and Hank’s BSS from PAA Laboratories (Coelbe, Germany), insulin-transferrin-selenium supplement from Gibco (Karlsruhe, Germany), fetal calf serum (FCS) from PAN Biotech (Aidenbach, Germany), triiodothyronine from Fluka (Buchs, Switzerland), hydrocortisone from Sigma (Munich, Germany), epidermal growth factor from PeproTech (Hamburg, Germany), TGF-β1 from tebu-bio (Offenbach, Germany), lysophosphatidic acid (LPA) (Sigma-Aldrich, Munich, Germany); Rho kinase inhibitor Y27632, TGF-β RI Kinase Inhibitor VI, SB431542, MEK kinase inhibitor U0126, p38 kinase inhibitor and brefeldin A were obtained from Calbiochem, Munich, Germany.

### Cell culture

Human primary tubular epithelial cells (hPTEC) were isolated from renal cortical tissues collected from healthy parts of tumor-nephrectomies essentially as described previously [18]. Isolation of human cells was approved by the local ethics committee and written consent was obtained from all donors. In brief, after transport in Hank’s BSS, cortex tissue was cut into 1 mm^3^ pieces and digested with collagenase type II (Gibco, Karlsruhe, Germany) and DNase I grade II (Roche Diagnostics, Mannheim, Germany) for 60 min. Next, cell suspension was sieved through 100 μm and 70 μm meshes. After a washing step with HBSS, cells were seeded in epithelial cell selective medium (DMEM/Ham’s F12 medium containing 2 mM L-glutamine, 100 U/ml penicillin, 100 μg/ml streptomycin, insulin-transferrin-selenium supplement, 10 ng/ml epidermal growth factor, 36 ng/ml hydrocortisone and 4 pg/ml triiodothyronine) in the presence of 0.5% FCS. After 1–2 days, medium was replaced by FCS-free medium. Cells were subcultured by application of trypsin. For experiments, hPTEC were seeded in medium containing 2.5% FCS to facilitate cell attachment, and medium was replaced after 24 h to FCS-free epithelial cell selective medium. Bright field pictures were recorded by Olympus CK40 microscope (Olympus, Hamburg, Germany) using Leica DC Viewer software (Leica, Herbrugg, Switzerland).

About five days after isolation at passage one cells were routinely analyzed for the content of proximal and distal cell by staining for N- and E-cadherin, respectively. The ratio of distal and proximal cells varied with most preparation containing a higher percentage of distal cells (about 70%).

Polarized tubular epithelial cells were obtained by culturing primary epithelial cells for 8 days on permeable transwell inserts (Millicell PCF, Millipore, Schwalbach, Germany).

### Transepithelial electrical resistance (TEER)

Transepithelial electrical resistance was measured using a commercially available epithelial vpltohmeter and a set of so-called chopstick electrodes (EVOM, World Precision Instruments, Berlin, Germany). All measurements were corrected for resistance of culture medium and empty inserts.

### Western blot analysis

Cell culture supernatants were precipitated with ethanol. Western blot analyses were performed essentially as described before [20] using the following antibodies: CTGF (SC-14939, Santa Cruz), phospho-ERK 1/2 and ERK 1/2 (Cell Signaling), and fibronectin HNF7.1 developed by R.J Klebe obtained from the Developmental Studies Hybridoma Bank developed under the auspices of the NICHD and maintained by The University of Iowa, Department of Biology, Iowa City, IA 52242. The immunoreactive bands were quantified using the luminescent image analyzer (LAS-1000 Image Analyzer, Fujifilm, Berlin, Germany) and AIDA 4.15 image analyzer software (Raytest, Berlin, Germany).

### Real time RT-PCR

RNA of cells cultured in inserts was isolated and analyzed as described previously [20].

### Immunocytochemistry

Cells were fixed with paraformaldehyde (3.5% in PBS) for 10 min and afterwards permeabilized by 0.5% Triton X-100 in PBS for 10 min. After washing three times with PBS, cells were blocked in 1% BSA in PBS. Antibodies used for Immunocytochemistry: E-cadherin (Abcam), N-cadherin (Santa Cruz), CTGF (Santa Cruz), acetylated tubulin (Sigma), Smad2/3 (Cell Signaling). Alexa Fluor 488- or 555-conjugated secondary antibodies were from Invitrogen.

After mounting, slides were viewed using a Nikon Eclipse 80i fluorescent microscope and digital images recorded by Visitron Systems 7.4 Slider camera (Diagnostic Instruments, Sterling Heights, MI, USA) using Spot Advanced software (Diagnostic Instruments). Three dimensional images were evaluated by epifluorescence microscopy including Apotome technique (Zeiss, Göttingen, Germany).

### Recombinant CTGF

A full length human CTGF cDNA, kindly provided by M. Bauer, Med. Clinic 1, Erlangen, was expressed in HEK293 cells. His-tagged CTGF was purified from cell culture supernatants using Ni-columns (Protino, Machery and Nagel, Düren, Germany). Purity of the protein was shown by silver staining of SDS polyacrylamide gels and estimated to be over 90%.

### Statistical analysis

To compare multiple conditions, statistical significance was calculated by one-way ANOVA with Dunnett’s post hoc test using GraphPad software. A value of P < 0.05 was considered to indicate significant differences.

## Abbreviations

hPTEC: Human Primary Tubular cells; LPA: Lysophosphatidic Acid; TEER: Transepithelial Electrical Resistance; TGF-β: Transforming Growth Factor Beta; UUO: Unilateral Ureter Obstruction.

## Competing interests

The authors declare that they have no competing interests.

## Authors’ contributions

JZ and AE performed the experiments, BK analyzed data and wrote the manuscript; MGS designed the study, analyzed data, drafted and wrote the manuscript. All authors read and approved the final version of the manuscript.
